# Supplementary material for: Ophthalmic nurses’ knowledge, attitude, and practice toward venous thromboembolic prevention: a dual-center cross-sectional survey
Source: PeerJ. 2023 Aug 28;11:e15947. doi: 10.7717/peerj.15947 (PMC10470452; doi:10.7717/peerj.15947)
Supplement: Supplemental Information 3 [file peerj-11-15947-s003.docx]

**Survey of ophthalmic nurses' knowledge, attitudes, and practice toward venous thromboembolic prevention**

Dear nursing colleagues:

Hello! Thanks for your participation in this survey. Venous thromboembolism (VTE) is an important cause of unexpected death and perioperative death in hospitals. The purpose of this study was to understand the level of knowledge, attitudes, and practice toward VTE prevention among ophthalmic nurses, and to provide a basis for further VTE prevention and improvement of clinical nursing quality. This questionnaire is anonymous and only for scientific research and analysis.

All your information will be confidential. Please answer according to the actual situation, sincerely thank you for your help and support!

Do you agree to participate in this study?

A. Yes

B. No

Signature: Date:

**Socio-demographic information**

1. Gender: A. Male B. Female
2. Age: years
3. Province

4. Hospital level: A. Tertiary hospital B. Secondary hospital C. First-level hospital

1. Hospital type: A. General hospital B. Specialized hospital

6. Service years: A. ≤ 5 B. 6-10 C. 11-15 D. ≥ 16

7. Professional title: A. Primary B. Intermediate C. Senior

8. Highest education attained:

A. Associate degree and below B. Bachelor’s degree C. Master’s degree and above

9. Position: A. Nurse B. Head nurse

10. Department: A. Inpatient department B. Outpatient department C. Operating room

11. Have you received VTE prevention training: A. Yes B. No

12. Your knowledge of VTE prevention is mainly obtained through the following ways: (multiple choice)

AInternet. Hospital or department training

B. Out-of-hospital training or academic conferences

C. Guideline or literature reading

D. Internet

E. School education

F. Other

13. Does your hospital carry out VTE prevention and control management:

A. Yes B. No

14. Whether VTE occurred during hospitalization in patients admitted to your department: A. Yes B. No

**Venous thromboembolism (VTE) related knowledge**

K1. VTE comprise deep vein thrombosis (DVT) and pulmonary embolism (PE).

1. True
2. False
3. Don't know

K2. Dyspnea, chest pain, syncope and shock are the main clinical manifestations of acute PE.

1. True
2. False
3. Don't know

K3. The main causes of VTE are venous stasis, blood hypercoagulability and vascular wall injury.

1. True
2. False
3. Don't know

K4. Post-thrombotic syndrome is the most severe complication of DVT.

1. True
2. False
3. Don't know

K5. DVT often occurs in the lower limbs.

1. True
2. False
3. Don't know

K6. After acute DVT of the legs, the patient 's lower limbs will have symptoms such as unilateral leg pain, redness, swelling, edema, warmth, and tenderness.

1. True
2. False
3. Don't know

K7. Compression ultrasonography is the first-line imaging test for patients with a suspected first DVT.

1. True
2. False
3. Don't know

K8. The Caprini risk assessment model of high VTE risk partition is 3 ~ 4 points.

1. True
2. False
3. Don't know

K9. Raising the lower limbs after surgery has no effect on preventing VTE.

1. True
2. False
3. Don't know

K10. Encouraging early ambulation after surgery may prevent VTE when the condition allows.

1. True
2. False
3. Don't know

K11. Giving up smoking and drinking has no effect on preventing VTE.

1. True
2. False
3. Don't know

K12. Avoiding venipuncture of lower limbs is ineffective in preventing VTE.

1. True
2. False
3. Don't know

K13. Physical methods to prevent VTE include graduated compression stockings, intermittent pneumatic compression devices and plantar venous pumps.

1. True
2. False
3. Don't know

K14. Physical prevention of VTE can be used for patients with congestive heart failure and severe edema of lower limbs.

1. True
2. False
3. Don't know

K15. When measuring the circumference of the lower limbs of the patients, the skin ruler, location and time should be monitored.

1. True
2. False
3. Don't know

K16. Ankle pump exercise can effectively prevent DVT.

1. True
2. False
3. Don't know

K17. Patients with DVT of lower limbs need to massage or hot compress the affected limb.

1. True
2. False
3. Don't know

K18. Infection is the most common complication after using anticoagulants.

1. True
2. False
3. Don't know

K19. Older age is a risk factor for VTE.

1. True
2. False
3. Don't know

K20. Obesity is not a risk factor for VTE.

1. True
2. False
3. Don't know

K21. Bedridden is not a risk factor for VTE.

1. True
2. False
3. Don't know

K22. Recent surgery is a risk factor for VTE.

1. True
2. False
3. Don't know

K23. Pregnancy or puerperium is a risk factor for VTE.

1. True
2. False
3. Don't know

K24. Oral contraceptives or hormone replacement therapy are not risk factors for VTE.

1. True
2. False

Don't know

K25. Personal or family history of VTE is a risk factor for VTE.

1. True
2. False
3. Don't know

K26. Calf swelling or varicose veins are risk factors for VTE.

1. True
2. False
3. Don't know

K27. Active cancer is not a risk factor for VTE.

1. True
2. False
3. Don't know

**Venous thromboembolism ( VTE ) related attitudes**

A1. Do you think VTE will increase the risk of nursing and even lead to medical disputes?

A. Strongly agree

B. Agree

C. Uncertainty

D. Disagree

E. Strongly disagree

A2. Do you think VTE is preventable?

A. Strongly agree

B. Agree

C. Uncertainty

D. Disagree

E. Strongly disagree

A3. Do you think that nurses should carry out health education on VTE prevention for patients and their families?

A. Strongly agree

B. Agree

C. Uncertainty

D. Disagree

E. Strongly disagree

A4. Do you think it is necessary to carry out VTE prevention and management in ophthalmic ward?

A. Strongly agree

B. Agree

C. Uncertainty

D. Disagree

E. Strongly disagree

A5. Do you think it is necessary to train ophthalmic nurses on the prevention and treatment of venous thrombosis?

A. Strongly agree

B. Agree

C. Uncertainty

D. Disagree

E. Strongly disagree

**Venous thromboembolism ( VTE ) related behavior**

P1. Can you use the VTE risk assessment scale properly?

1. Always
2. Often
3. Sometimes
4. Occasionally
5. Never

P2. Will you conduct a VTE risk assessment of inpatients with eye diseases?

1. Always
2. Often
3. Sometimes
4. Occasionally
5. Never

P3. When the patient's assessment result is a high risk of VTE, will you notify the doctor promptly?

1. Always
2. Often
3. Sometimes
4. Occasionally
5. Never

P4. Will you assess the high risk patients of VTE regularly?

1. Always
2. Often
3. Sometimes
4. Occasionally
5. Never

P5. Will you hand over patients with a high VTE risk in bedside-shift?

1. Always
2. Often
3. Sometimes
4. Occasionally
5. Never

P6. Will you carry on health education about VTE prevention for patients and their families?

1. Always
2. Often
3. Sometimes
4. Occasionally
5. Never

P7. Will you give timely feedback to the doctor about the suspicious symptoms of VTE?

1. Always
2. Often
3. Sometimes
4. Occasionally
5. Never

P8. Do you assess the lower limbs of perioperative patients regularly in nursing practice?

1. Always
2. Often
3. Sometimes
4. Occasionally
5. Never

P9. If the patient's condition allows, do you encourage them early ambulation after eye surgery?

1. Always
2. Often
3. Sometimes
4. Occasionally
5. Never

P10. When the patient has DVT, will you regularly dynamically observe and record the swelling, pain, and calf circumference of the affected limb?

1. Always
2. Often
3. Sometimes
4. Occasionally
5. Never

P11. When a patient has DVT, do you regularly observe whether the patient has PE symptoms such as dyspnea, chest pain, syncope, tachycardia, and hypotension?

1. Always
2. Often
3. Sometimes
4. Occasionally
5. Never

P12. When patients use anticoagulants, will you monitor the effectiveness and side effects of drugs?

1. Always
2. Often
3. Sometimes
4. Occasionally
5. Never
