# Supplementary material for: Ophthalmic nurses’ knowledge, attitude, and practice toward venous thromboembolic prevention: a dual-center cross-sectional survey
Source: PeerJ. 2023 Aug 28;11:e15947. doi: 10.7717/peerj.15947 (PMC10470452; doi:10.7717/peerj.15947)
Supplement: Supplemental Information 4 [file peerj-11-15947-s004.doc]

眼科护士静脉血栓栓塞症预防知识、态度及行为调查

尊敬的各位护理同仁：

您好！感谢您在百忙之中参与本次调查。静脉血栓栓塞症（VTE）是医院内非预期死亡及围手术期死亡的重要原因。本研究旨在了解眼科护士对VTE预防与护理知信行水平，为进一步开展VTE防治及提升临床护理质量提供依据。本问卷为匿名填写，仅供科研分析，您的所有信息我们均将保密。请您根据实际情况回答即可，真诚感谢您的帮助与支持！

您是否知情同意，愿意参加本研究？

1. 是
2. 否

签名： 日期：

**一、一般资料**

1. 性别： A. 男 B. 女
2. 年龄： 岁
3. 省 市
4. 医院等级： A. 三级 B. 二级医院 C. 一级医院
5. 医院种类： A. 综合医院 B. 专科医院
6. 工作年限： A. ≤5 B. 6-10 C. 11-15 D. ≥16
7. 职称: A. 初级（护士和护师） B. 中级（主管护师） C. 高级（副主任护师及以上）
8. 最高学历： A. 大专及以下 B. 本科 C. 硕士及以上
9. 职务： A. 无 B. 护士长
10. 工作岗位： A. 病房护士 B. 门诊护士 C. 手术室护士
11. 您是否接受过VTE相关知识的培训：A. 是 B. 否
12. 您的VTE防治知识主要通过以下哪些途径获得：（多选题）
13. 院内或科室培训
14. 院外培训或学术会议
15. 阅读指南或文献
16. 网络平台（如微信公众号）
17. 学校教育
18. 其他
19. 您所在医院是否开展VTE防治管理：A. 是 B. 否（跳转至14题）
20. 您科室收治的患者，住院过程中是否发生过VTE：A. 是 B. 否
21. **静脉血栓栓塞症（VTE）相关知识**
22. 静脉血栓栓塞症包括深静脉血栓形成（DVT）和肺血栓栓塞症（PTE）。

对

错

不知道

1. 呼吸困难、胸痛、晕厥和休克是急性肺栓塞的主要临床表现。

对

错

不知道

1. VTE形成的主要原因是血流瘀滞、血管壁损伤和血液高凝状态。

对

错

不知道

1. 深静脉血栓形成最严重的并发症是血栓后综合征。

对

错

不知道

1. 深静脉血栓形成常发生于下肢静脉。

对

错

不知道

1. 急性下肢深静脉血栓形成后，患者下肢会出现肿胀、压痛、皮色泛红、皮溫升高等症状。

对

错

不知道

1. 怀疑下肢深静脉血栓首选彩色多普勒超声进行辅助诊断。

对

错

不知道

1. Caprini量表评分具有VTE高度风险的分值是3～4分。

对

错

不知道

1. 术后抬高下肢，对预防VTE无效。

对

错

不知道

1. 当病情允许时，鼓励患者早期床上或下床活动可预防VTE。

对

错

不知道

1. 戒烟、戒酒、控制血脂和血糖对预防VTE无效。

对

错

不知道

1. 避免下肢静脉穿刺及患肢制动侧穿刺对预防VTE无效。

对

错

不知道

1. 预防VTE的物理方法包括梯度压力弹力袜、间歇式充气加压装置和足底静脉泵。

对

错

不知道

1. 下肢深静脉血栓形成的物理预防方法适用于充血性心力衰竭和下肢严重水肿的患者。

对

错

不知道

1. 对患者进行下肢周径测量时需定皮尺、定部位、定时间监测。

对

错

不知道

1. 踝泵运动能有效预防深静脉血栓。

对

错

不知道

1. 下肢深静脉血栓形成的患者需对患肢进行按摩或热敷。

对

错

不知道

1. 感染是使用抗凝药物后最常见的并发症。

对

错

不知道

1. 高龄是VTE发生的危险因素。

对

错

不知道

1. 肥胖不是VTE发生的危险因素。

对

错

不知道

1. 长期制动或卧床不是VTE发生的危险因素。

对

错

不知道

1. 近期手术是VTE发生的危险因素。

对

错

不知道

1. 妊娠期或产后1个月内是VTE发生的危险因素。

对

错

不知道

1. 口服避孕药或激素替代治疗不是VTE发生的危险因素。

对

错

不知道

1. VTE病史或家族史是VTE发生的危险因素。

对

错

不知道

1. 下肢水肿或下肢静脉曲张是VTE发生的危险因素。

对

错

不知道

1. 恶性肿瘤不是VTE发生的危险因素。

对

错

不知道

1. **静脉血栓栓塞症（VTE）相关信念**
2. 您认为VTE会增加护理风险，甚至引发医疗纠纷吗？ VTE will increase nursing risk and even cause medical disputes.
3. 非常同意
4. 同意
5. 不确定
6. 不同意
7. 非常不同意
8. 您认为VTE是可以预防的吗？VTE is preventable.
9. 非常同意
10. 同意
11. 不确定
12. 不同意
13. 非常不同意
14. 您认为护理人员承担着患者及家属预防静脉血栓的健康教育任务吗？Nurses should carry out health education on VTE prevention for patients and their families.
15. 非常同意
16. 同意
17. 不确定
18. 不同意
19. 非常不同意
20. 您认为在眼科病房开展VTE防治管理的必要性？The prevention of VTE in ophthalmic ward is very important.
21. 非常有必要
22. 有必要
23. 不确定
24. 没必要
25. 非常没必要
26. 您认为对眼科护理人员进行静脉血栓防治相关知识培训的必要性？It is necessary to train nurses in VTE prevention.
27. 非常有必要
28. 有必要
29. 不确定
30. 没必要
31. 非常没必要

**四、静脉血栓栓塞症（VTE）相关行为**

1. 您能正确使用VTE风险评估表对患者进行风险评估吗？

总是

经常

有时

偶尔

从不

1. 您会对眼病住院患者进行VTE风险评估，筛选出高危患者吗？

总是

经常

有时

偶尔

从不

1. 当患者评估结果为VTE高危风险时，您会及时通知医生吗？

总是

经常

有时

偶尔

从不

1. 您会对具有高危VTE风险的眼病患者进行动态评估吗？

总是

经常

有时

偶尔

从不

1. 您会与下一班护士交接具有VTE形成高风险的患者情况吗？

总是

经常

有时

偶尔

从不

1. 您会对眼病住院患者或家属进行预防VTE的健康宣教吗？

总是

经常

有时

偶尔

从不

1. 您会向医生及时反馈患者发生VTE的可疑症状吗？

总是

经常

有时

偶尔

从不

1. 您在临床工作中会定时观察围手术期眼病患者的下肢情况吗（有无肿胀、压痛、皮溫、足背动脉搏动）？

总是

经常

有时

偶尔

从不

1. 病情允许的情况下，您指导过眼病手术后患者早期进行床上活动、被动活动吗？

总是

经常

有时

偶尔

从不

1. 当患者已发生DVT时，您会动态观察患肢的肿胀、疼痛、小腿周径等情况并记录吗？

总是

经常

有时

偶尔

从不

1. 当患者已发生DVT时，您会动态观察患者有无呼吸困难，胸痛等肺栓塞症状吗？

总是

经常

有时

偶尔

从不

1. 当患者使用抗凝药物时，您会及时评估药物的有效性及不良反应吗？

总是

经常

有时

偶尔

从不
